# Transgenerational effects of polychlorinated biphenyls: 1. Development and physiology across 3 generations of rats

**DOI:** 10.1186/s12940-018-0362-5

**Published:** 2018-02-20

**Authors:** Jan A. Mennigen, Lindsay M. Thompson, Mandee Bell, Marlen Tellez Santos, Andrea C. Gore

**Affiliations:** Division of Pharmacology and Toxicology, College of Pharmacy, University of Texas at Austin, 107 W Dean Keeton, C0875, Austin, TX 78712 USA

**Keywords:** Polychlorinated biphenyl (PCB), Reproduction, Estradiol, Progesterone, Corticosterone, Body weight, Endocrine-disrupting chemical (EDC), Transgenerational, Sex difference

## Abstract

**Background:**

Polychlorinated biphenyls (PCBs) are persistent organic environmental contaminants and known endocrine-disrupting chemicals (EDCs). Previous studies demonstrated that developmental exposure to the weakly estrogenic PCB mixture Aroclor 1221 (A1221) in Sprague-Dawley rats altered sexual development, adult reproductive physiology and body weight. The current study tested the hypothesis that prenatal A1221 exposure not only disrupts these endpoints within an exposed individual’s (F_1_ generation) lifespan, but may also affect subsequent generations (F_2_-F_3_).

**Methods:**

We treated pregnant female rats on embryonic days (E) 16 and E18 with A1221 (1 mg/kg), estradiol benzoate (50 μg/kg, positive estrogenic control), or vehicle (3% DMSO in sesame oil, negative control). Endpoints related to sexually dimorphic developmental trajectories of reproductive and developmental physiology were measured, and as adults, reproductive endocrine status was assessed, in the F_1_, F_2_, and F_3_ generations.

**Results:**

Significant effects of transgenerational EDCs were found for body weight and serum hormones. The A1221 descendants had significantly higher body weight in the F_2_-maternal lineage throughout postnatal development, and in F_3_-maternal lineage animals after weaning. In females, generation- and lineage-specific effects of exposure were found for serum progesterone and estradiol. Specifically, serum progesterone concentrations were lower in F_2_-A1221 females, and higher in F_3_-A1221 females, compared to their respective F_2_- and F_3_-vehicle counterparts. Serum estradiol concentrations were higher in F_3_-A1221 than F_3_-vehicle females. Reproductive and adrenal organ weights, birth outcomes, sex ratio, and estrous cycles, were unaffected. It is notable that effects of A1221 were only sometimes mirrored by the estrogenic control, EB, indicating that the mechanism of action of A1221 was likely via non-estrogenic pathways.

**Conclusions:**

PCBs caused body weight and hormonal effects in rats that were not observed in the directly exposed F_1_ offspring, but emerged in F_2_ and F_3_ generations. Furthermore, most effects were in the maternal lineage; this may relate to the timing of exposure of the F_1_ fetuses at E16 and 18, when germline (the future F_2_ generation) epigenetic changes diverge in the sexes. These results showing transgenerational effects of EDCs have implications for humans, as we are now in the 3rd generation since the Chemical Revolution of the mid-twentieth century, and even banned chemicals such as PCBs have a persistent imprint on the health of our descendants.

**Electronic supplementary material:**

The online version of this article (10.1186/s12940-018-0362-5) contains supplementary material, which is available to authorized users.

## Background

Polychlorinated biphenyls are a class of persistent organic pollutants consisting of 209 specific congeners that were heavily used by industry for decades before being banned in the late 1970s [1]. Today, due to their environmental persistence and high capacity for biomagnification in food chains, PCBs remain detectable in most wildlife and humans [2, 3]. Both experimental and epidemiological lines of evidence support endocrine-disrupting properties of specific PCB congeners and mixtures, with well-described consequences on reproductive [4–6] and metabolic [7, 8] physiology. More specifically, the weakly estrogenic PCB mixture Aroclor 1221 (A1221) disrupted sexual development and led to sex-specific effects on adult reproductive physiology and its neuroendocrine regulation, potentially through estrogenic mechanisms [9–11]. For body weight, recent studies have suggested obesogenic effects of A1221 that, in contrast, do not point to an estrogenic mode of action [12, 13].

Most work on EDCs has focused on their direct effects in animals exposed during early life development (F_1_ generation). However, evidence over the last decade has shown intergenerational effects of endocrine-disrupting chemicals (EDCs) in the F_2_ generation, and transgenerational effects in F_3_ generations and beyond [14–18], although little of this work was on PCBs [4, 19]. In addition, the question of transmission of disrupted traits across generations has been seldom studied in head-to-head comparisons of maternal vs. paternal lineages, which would likely lead to very different phenotypic outcomes due to sex differences in the timing of epigenetic programming [20].

In the current study, we sought to determine effects of the PCB mixture, A1221, on the F_1_, F_2_, and F_3_ generations of maternally- and paternally-derived lineages. The A1221 mixture has a relatively short half-life compared to most PCBs [21, 22] allowing us to target the exposure to a specific developmental window in exposed individuals. We administered the treatments during the beginning of the critical period of brain sexual differentiation, and conducted work on both females and males to enable us to discern sex differences in direct and inherited responses to environmental EDCs. This experimental approach is translationally relevant [23], given that the human generational time is 20–30 years; therefore, we are in the 3rd human generation following the chemical revolution in the mid-twentieth century. Indeed, living in a contaminated world in which animal and human species are incidentally exposed to persistent man-made chemicals such as PCBs even in remote areas, it becomes paramount to understand if and how physiology across development and generations is affected by environmental contaminants [24].

## Methods

### Experimental design and animal husbandry

**Fig. 1:**
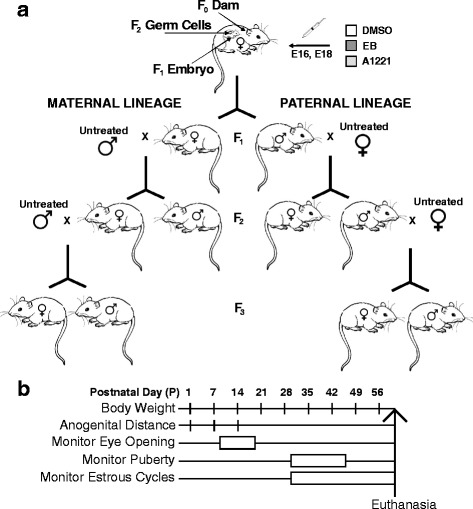
**a** The transgenerational experimental design and breeding strategy of the study. **b** The developmental timeline and endpoints for individual animals. Abbreviations: E: embryonic day of age

Female and male Sprague-Dawley rats were purchased from Harlan (Houston, TX), and all animal procedures were conducted in compliance with protocols approved by the Institutional Care and Use Committee at the University of Texas at Austin, and following NIH guidelines. Rats were housed in a colony room with controlled temperature (22 °C) and partially reversed light cycle (12:12 dark:light, lights on at 2400 h). Virgin females were mated with sexually-experienced males. The day following successful mating, as indicated by a sperm-positive vaginal smear, was termed embryonic day 1 (E1). During pregnancy, rats (termed F_0_) were exposed to one of three treatments, given on E16 and E18 to target the beginning of the period of sexual differentiation of the rat brain [25–27]. The treatments were: 1 mg/kg PCB mixture Aroclor 1221 (A1221, i.p.); 50 μg/kg estradiol benzoate (EB, s.c.) as a positive control for the estrogenic effects of A1221; or a negative vehicle control (3% DMSO in sesame oil; half of these animals received i.p., and half s.c., injections). The A1221 PCB mixture is lightly chlorinated, resulting in a relatively short biological half life in the range of days [21, 22]. The treatment dosages were based on prior work conducted in the Gore lab, which elicited developmental and adult neuroendocrine effects on reproductive physiology at multiple levels of biological organization [4, 5, 9, 10]. The day of parturition was referred to as postnatal day 0 (P0), and litters were culled to 3 males and 3 females per group or as close as possible until weaning, after which animals were housed in male- and female-specific sibling triads. Upon reaching sexual maturity, one exposed male and female offspring (F_1_) per litter were used as founders of paternal and maternal lineages of all treatment groups (F_2_), and mated with untreated adult rats. One F_2_ male from each paternal litter, and one female from each maternal litter were subsequently mated with untreated adult rats to extend paternal and maternal lineages into the F_3_ generation. Figure 1 summarizes the transgenerational experimental design (Fig. 1a), and an experimental timeline of individual measurements and sampling protocols (Fig. 1b**)**. Sample sizes and groups are in Table 1.

**Table 1:** Groups and sample sizes. Individual animal numbers per group are indicated, with numbers of litters in parentheses

Generation	Females		Males	
F_1_	DMSO: 41 (14) EB: 32 (11) A1221: 35 (12)		DMSO: 39 (14) EB: 32 (11) A1221: 36 (12)	
Generation and lineage	Maternal	Paternal	Maternal	Paternal
F_2_	DMSO:35 (12) EB: 32 (11) A1221:32 (11)	DMSO: 38 (14) EB: 30 (11) A1221: 35 (12)	DMSO: 34 (12) EB: 33 (11) A1221: 30 (11)	DMSO: 38 (14) EB: 32 (11) A1221: 34 (12)
F_3_	DMSO:33 (11) EB: 30 (11) A1221:31 (11)	DMSO: 39 (14) EB: 32 (11) A1221: 30 (11)	DMSO: 32 (11) EB: 30 (11) A1221: 27 (11)	DMSO: 39 (14) EB: 31 (11) A1221: 31 (11)

To provide breeder males with sexual experience, female stimulus rats were purchased as young adults from Harlan, and ovariectomized under isoflurane anesthesia. Stimulus animals were not subjected to any of the EDC treatments, although the ovariectomized females were primed with estradiol plus progesterone to induce receptivity. In addition, breeding male and female partners (untreated with EDCs or vehicle) were purchased to generate the F_2_ and F_3_ generations.

### Developmental endpoints

On P1, the day after birth, pups were weighed and their anogenital distance (AGD) was measured using a digital microcaliper. The anogenital index (AGI) was calculated (AGI = AGD divided by the cube root of body weight) as it enables proper sexing of neonates and also serves as an indicator of masculinization or feminization [28]. Characteristics of birth outcomes (number of male and female pups and sex ratio were determined; they were unaffected by treatment (Additional file 1: Figure S1). Litters were culled as follows: the three males and females with AGIs closest to the median of the same sex and litter were kept to achieve equal litter size and sex ratio. All litters, including controls, were culled to a total of 6 pups (3 male and 3 female) to ensure that sex ratio was equal, to avoid possible confounding effects of a biased sex ratio, and to allow us to attain adequate statistical power based on a power analysis. Litter was considered as a variable in analyses (below). Along with being measured on P1, AGI was monitored weekly on P7 and 14 (Fig. 1b). Age at eye opening was assessed before weaning, and time of puberty was assessed after weaning in females (day of vaginal opening) and males (day of preputial separation). Body weight was recorded weekly from P1–56 (Fig. 1b).

### Monitoring of estrous cycles

Following puberty in females, daily vaginal smears were taken to track estrous cycles by vaginal cytology under a light microscope. Based on these data, several parameters were determined: The number of completed estrous cycles from puberty to age at P56, average length of estrous cycles in days, and percentage of elongated (duration >5d) or irregular (aberrant transitions) cycles were determined. No significant treatment effects were found on these endpoints (Additional file 2: Figure S2).

### Tissue collection

Within each generation (F_1_-F_3_), animals were euthanized by rapid decapitation 2–3 h before lights-out. To minimize the possible confound of the estrous cycle on hormone concentrations, females were euthanized on proestrus as close to P60 as possible, between P58–63, and for each female, a male littermate was euthanized on the same day. Thus all rats were euthanized from P58–63. Trunk blood was collected, allowed to clot, and serum was separated via centrifugation (1500×*g* for 5 min). Tissues and sera were stored at − 80 **°**C until use. Dams of all generations were euthanized between 1 and 2 weeks after pups were weaned in order to examine the uterus for the number of implantation sites, and to calculate the number of resorptions based on the difference between numbers of pups born and implantation sites. No effects of treatment on resorptions was observed (Additional file 1: Figure S1).

### Hormone assays

Serum samples were used to determine concentrations of circulating estradiol, progesterone, testosterone, and corticosterone using previously validated commercial kits [12, 13, 29]. The assays and their characteristics were: estradiol, Beckman #DSL-4800, range 5–750 pg/ml, sensitivity 2.2 pg/ml; progesterone, Cayman #582601, range 7.8–1000 pg/ml, sensitivity 10 pg/ml; testosterone, MP Biomedical #07189102, range 0.1–10 ng/ml, sensitivity 0.03 ng/ml; and corticosterone, MP Biomedical #07120102, range 25–1000 ng/ml, sensitivity 7.7 ng/ml. Final sample sizes for individual treatment groups within sex and lineage were *n* = 17–25 for estradiol_,_ *n* = 8–10 for progesterone, *n* = 10 for testosterone (males only, as the assay is not sensitive enough to measure testosterone in females) and n = 10 for corticosterone. All assays were run according to the manufacturers’ protocols.

### Statistical analyses and graphs

The identity of treatment groups and sample numbers were coded throughout the study, in order to ensure that for experimenters were blind to the treatment in the analyses of all endpoints*.* For all datasets, a custom R-based, generalized extreme studentized deviate (ESD) test was used to detect outliers, limited to a maximum of two per group. Following the validation of normality and homoscedasticity in groups using Shapiro-Wilk and Levene’s test, respectively, data were analyzed using a general linear model approach. A two-way ANOVA was used for F_1_ data, with sex and treatment as factors, and litter as random factor. For data from F_2_ and F_3_ animals, a three-way ANOVA, with sex, lineage, and treatment as main factors, and litter as random factor, was used. When data were non-parametric after standard transformation procedures, Kruskal-Wallis analysis was used for individual factors. Repeated measures tests (split-plot ANOVAs) were used for longitudinal datasets (body weight and AGI), using time as factor within groups. In cases of repeated developmental data measurements, Greenhouse-Geisser corrections for violations of sphericity (assessed by Mauchly’s test), were used where appropriate. Data that revealed significant effects of treatment or treatment interactions with other analyzed factors were further analyzed using Sidak’s post-hoc test, in order to determine global treatment differences or treatment differences within interacting factors. In cases where the data did not satisfy the assumptions for parametric analyses, post-hoc analysis was conducted using Dunn’s test. Post-hoc comparisons of significant treatment effects in repeated measures ANOVAs were analyzed using Sidak’s post-hoc comparisons. While descriptive statistics and outlier identification was conducted using a custom R scripts, general linear model analyses were conducted using SPSS Version 24 (IBM, New York, NY, USA). Graphs were plotted using Prism Version 7 (Graphpad, LaJolla, CA, USA). For the sake of presentation, detailed statistical outcomes (F-, H- and *p*-values, and degrees of freedom) are described only for significant treatment differences (*p* < 0.05); however all detailed model parameters can be accessed in Additional file 3: Figure S3.

## Results

### Prenatal A1221 effects on body weight

**Fig. 2:**
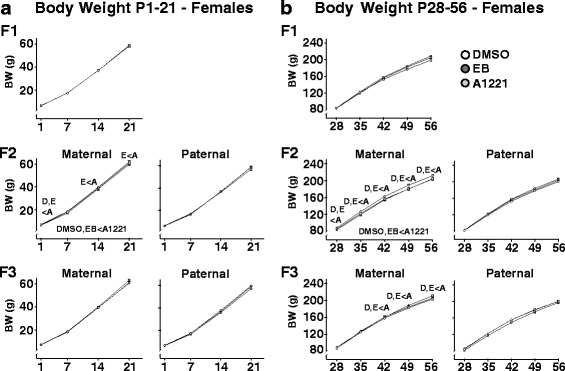
Developmental profiles of female body weight are shown for pre-weaning rats from P1–21 (**a**), and for adolescents from P28–56 (**b**), across the three generations. Data were analyzed by repeated measure ANOVAs, and significant overall treatment effects between groups (*p* < 0.05) are indicated within the panels below the graphs. In cases of significantly different treatment, lineage, and time interactions, specific differences for each time point were resolved by Sidak-adjusted post-hoc comparisons, indicated above individual time points. For the latter, the treatment groups are abbreviated as D, DMSO; E, EB; A, A1221

**Fig. 3:**
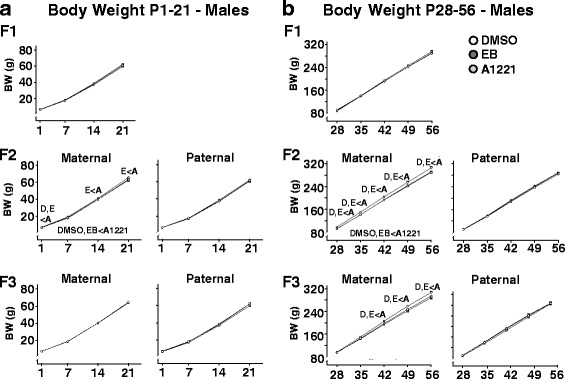
Developmental profiles of male body weight are shown for pre-weaning rats from P1–21 (**a**), and for adolescents from P28–56 (**b**), across the three generations. Data were analyzed, and abbreviations are the same, as in Fig. 2

Body weight was monitored weekly from P1–56, and due to the large sexual dimorphism in this endpoint across all generations (Additional file 3: Figure S3), data are depicted separately for females (Fig. 2) and males (Fig. 3). Data were further subdivided into two developmental periods: pre-weaning (P1–21) and adolescent (P28–56), due to the developmental shift in the body weight curve trajectory post-weaning, as reported previously [13]. In F_1_ animals, no significant treatment effects were observed (Additional file 3: Figure S3). Lineage-dependent treatment effects were detected in F_2_ animals (F_2.83, 3616_ = 7.77, *p* < 0.01), and Sidak’s post-hoc test revealed an overall significant increase in body weight in the A1221 group compared to DMSO and EB groups in maternal lineage descendants, irrespective of sex (*p* < 0.05). An interaction of treatment and lineage was also observed over time (F_2.83, 3616_ = 3.41, *p* < 0.01), and Sidak-adjusted post-hoc comparisons of lineage-dependent treatment effects at individual time points revealed that in the maternal lineage of the F_2_ generation (both males and females), ancestral A1221 treatment significantly increased body weight compared to DMSO and EB at P1 and for all time points following P28 (*p* < 0.05). In F_3_ animals, a significant interaction of treatment and lineage was observed over time (F_2.83, 3466_ = 3.48, p < 0.01), and Sidak-adjusted post-hoc comparisons of lineage-dependent treatment effects at individual time points revealed that in the maternal lineage of the F_3_ generation, ancestral A1221 treatment significantly increased bodyweight compared to DMSO and EB (*p* < 0.05) from P42 to P56.

### Prenatal A1221 exposure had few effects on developmental milestones of eye opening and puberty

**Fig. 4:**
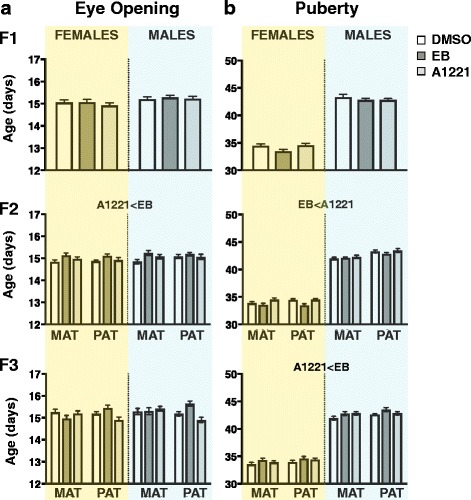
Developmental milestones of age at eye opening (**a**) and puberty (**b**) are shown for female and male rats of the three generations. Puberty in females was assessed by the day of vaginal opening, and in males, the day of preputial separation. Here and in subsequent figures that include data from both sexes, data from female are shaded yellow, and males in blue, to improve visualization. Significant (p < 0.05) treatment effects are indicated. Data were analyzed using the Kruskal-Wallis test. Abbreviations: MAT, maternal; PAT, paternal

Age at eye opening (Fig. 4a) was sexually dimorphic in F_1_ (H_1,215_ = 4.49, p < 0.05) and F_2_ (H_1,403_ = 3.92, p < 0.05), but not F_3_ animals (H_1,385_ = 3.24, *p* > 0.05). Prenatal treatment did not affect postnatal age of eye opening in F_1_ animals (H_2,215_ = 1.17, p > 0.05). In F_2_ rats, eye opening exhibited a significant difference between treatments (H_2,202_ = 6.89, *p* < 0.01), with a delay in EB-lineage animals compared to A1221 (*p* < 0.05). F_3_ animals did not exhibit significant treatment effects on age at eye opening (H_2_,_385_ = 2.653, p > 0.05).

Markers of the onset of puberty (vaginal opening in females, preputial separation in males; Fig. 4b) occurred significantly earlier in females compared to males in all generations (F_1_: H_1,215_ = 659, *p* < 0.01; F_2_: H_1,403_ = 1422, *p* < 0.01; F_3_: H_1,385_ = 1181; p < 0.01). No treatment effects were found in F_1_ animals (H_1,215_ = 0.78; p > 0.05). In F_2_ animals, there was a significant treatment effect on puberty (H_2,202_ = 5.44, p < 0.01), attributable to EB rats having significantly earlier puberty than A1221 rats. In the F_3_ generation, puberty onset was also affected by treatment (H_1,385_ = 4.427, *p* < 0.05), with EB rats displaying later puberty compared to A1221 treated rats (p < 0.05).

### Anogenital index was affected by EB in F_1_, and by A1221 and EB in F_3_ maternal lineage descendants

**Fig. 5:**
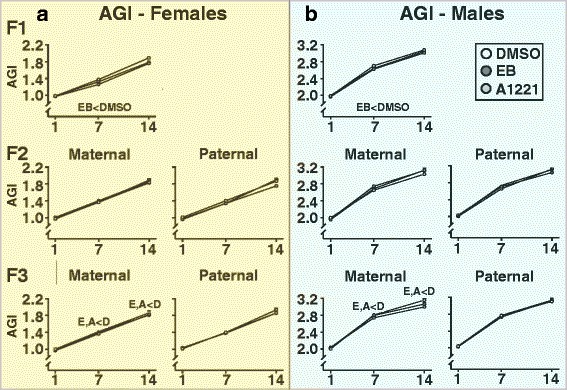
Anogenital index (AGI), calculated as [anogenital distance / ^3^√ body weight] is shown for females (**a**) and males (**b**) of the three generations. Data were analyzed using repeated measures ANOVAs. Significant overall treatment effects between groups (p < 0.05) are indicated within the panels below the graphs. In cases of significantly different main effects of treatment between groups, specific differences for each time point were resolved by Sidak-adjusted comparisons, indicated above individual time points

The expected strong sexual dimorphism of AGI (Fig. 5a, b) was found in all 3 generations (F_1_: F_1,215 =_ 226, p < 0.01; F_2_: F_1,202_ = 1054, p < 0.01; F_3_: F_1,385_ = 11,013, p < 0.01). Treatment significantly affected F_1_ generation AGI (F_2,324_ = 9.55, p < 0.01), with the EB rats having smaller AGIs than their DMSO counterparts (*p* < 0.05). In F_3_ animals, we found treatment effects to be lineage-specific, as evidenced by a significant interaction of both factors (F_2,385_ = 3.37, p < 0.05). Sidak’s post-hoc test reveals the DMSO group had an overall higher AGI compared to the A1221 group within the maternal lineage (p < 0.05). A significant interaction of treatment and lineage was also evident over time in F_3_ animals (F_1155, 0.257_ = 3.571, p < 0.05), and Sidak-adjusted post-hoc comparisons revealed significantly lower AGI in EB and A1221 groups (p < 0.05) compared to DMSO group animals in the maternal F_3_ lineage at P7 and P14.

### A1221 treatment did not affect adult endocrine tissue weights

**Fig. 6:**
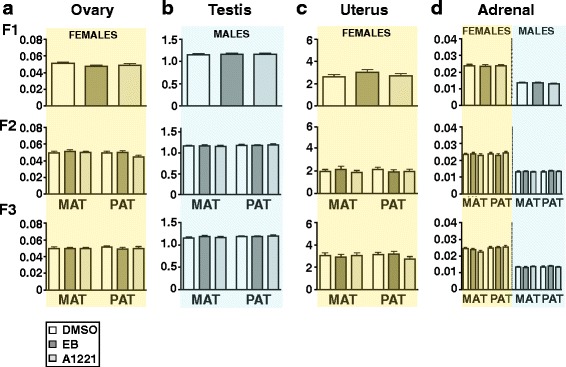
Endocrine tissue indices (organ weight normalized to body weight) are shown for the ovary (**a**), testis (**b**), uterus (**c**), and adrenal (**d**). Data were analyzed by univariate one-way ANOVAs. A significant sexual dimorphism in adrenal index was found across the 3 generations. No treatment effects were observed. Abbreviations: MAT, maternal; PAT, paternal

The gonadal, uterine, and adrenal indices were calculated by normalizing organ weight to body weight. There was no main effect of treatment, nor any interaction effects, on any of these organ indices in any generation (Fig. 6). The expected sexual dimorphism in the adrenal index was found throughout the generations (female > male). Detailed statistical analysis results are described in Additional file 3: Figure S3.

### Ancestral A1221 exposure altered estradiol and progesterone concentrations in females only, in a generation- and lineage-dependent manner

**Fig. 7:**
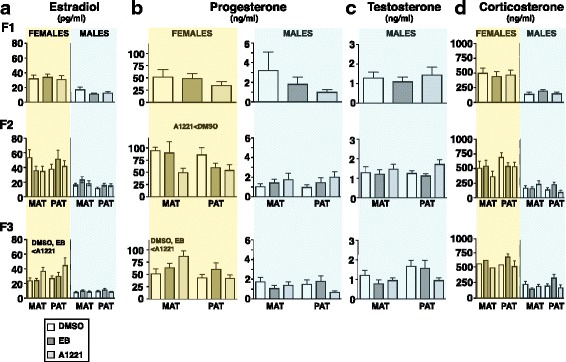
Serum hormone concentrations are shown in adult (~P60) female and male rats across the three generations for estradiol (**a**), progesterone (**b**), testosterone (**c**), and corticosterone (**d**). Note that serum progesterone concentrations are shown on different y-axes for the sexes; estradiol and corticosterone are plotted on the same y-axis scale for both sexes. To more easily visualize data, yellow shading shows results from females, and blue from males. Data were analyzed by univariate one-way ANOVAs. In cases of significantly different main effects of treatment between groups (p < 0.05), specific differences for each time point have been resolved by Tukey’s post-hoc comparisons. Abbreviations: MAT, maternal; PAT, paternal

The expected sex differences were found for the steroid hormones estradiol, progesterone, and corticosterone in all generations (Additional file 3: Figure S3). For serum estradiol concentrations (Fig. 7a), there were no effects of treatment in females of the F_1_ or F_2_ generation. However, in F_3_ females, a significant interaction of treatment and sex was found (F_2,129_ = 5.14, *p* < 0.05), irrespective of lineage. Post-hoc analysis revealed that in F_3_ females, serum estradiol was significantly higher in A1221 compared to DMSO and EB rats (p < 0.05).

For serum progesterone concentrations (Fig. 7b), the expected sexual dimorphism was found (female > male). F_1_ animals were not affected by treatment. F_2_ animals had a significant treatment effect dependent on sex (F_2,55_ = 3.517, p < 0.05), with female A1221 rats having lower progesterone concentrations than DMSO controls (p < 0.05), irrespective of lineage. In F_3_ animals, a significant treatment effect dependent on lineage and sex (F_1_,_58_ = 3.23, p < 0.05, Fig. 7b) was found. In the maternal lineage females, serum progesterone was significantly higher in A1221 compared to DMSO and EB descendants (p < 0.05). There was no treatment effect in the F_3_-paternal lineage females. In males, treatment did not affect serum progesterone concentrations in any generation (Additional file 3**:** Figure S3).

Serum concentrations of testosterone in males (Fig. 7c; female testosterone could not be measured due to limits of assay detection), and corticosterone in both sexes (Fig. 7d), were not affected by treatment. The expected sexual dimorphism of corticosterone (females > males) was confirmed in this study (Additional file 3**:** Figure S3), and was consistent with the larger adrenal indices in the females (Fig. 6d).

## Discussion

Exposures to endocrine-disrupting chemicals (EDCs) during critical periods of development have been linked to changes in reproductive, metabolic, and other endocrine functions later in life, consistent with the “developmental origins of health and disease” (DOHAD) hypothesis [30–32]. While much work has focused on outcomes in the exposed F_1_ generation, inter-generational (F_2_) and transgenerational (F_3_) effects are beginning to be demonstrated for low-dose EDC exposures [16, 17]. The expectation is that the phenotype will be unique to each generation due to the differential timing of exposure [33, 34], as follows. The F_1_ generation is exposed directly as embryos on E16 and E18, the beginning of the critical period of brain sexual differentiation in rats [25–27] and a life stage during which the entire body undergoes enormous developmental change. The F_2_ generation is exposed as germ cells within the F_1_ embryos. During germline development, epigenetic modifications of DNA demethylation and remethylation, histone modifications, and non-coding RNAs take place [35–37]. This epigenetic programming could lead to phenotypic changes in the F_2_ generation that are likely to be quite different from that of their F_1_ progenitors. Furthermore, the timing of demethylation and remethylation is not identical in the sexes [20, 38–40], leading to the expectation of sexually-dimorphic transgenerational effects. For the F_3_ generation, any exposure effects are truly transgenerational [33, 41], and must be inherited via germline or context-dependent (e.g. maternal behavior) mechanisms [42]. Again, the expectation is that the F_3_ generation will differ from both F_1_ and F_2_ generations; but that subsequent (F_4_ and beyond) generations would better phenocopy the F_3_ generation due to heritable epigenetic programming. This current study was our initial step to thoroughly document the inter- and transgenerational phenotype; a companion study will focus on specific neuroendocrine molecular outcomes.

One of our most interesting findings was a small but significant and consistent increase in body weight in A1221-lineage rats, restricted to the maternal-lineage F_2_ and F_3_ animals. This effect was not mimicked in the EB-descendants, suggesting a maternally transmitted, estrogen-independent mode-of action of A1221. While in the F_2_ generation this effect was detectable as early as P1, and maintained through adulthood, in the F_3_ generation the onset of this body weight difference did not take place until post-weaning. The dramatically different diet and energetic demands of an individual pre- vs. post-weaning may account for the adolescent onset; rat pups continue to nurse through P21 and begin to eat chow at ~P18 in our colony. After P21, rats are limited entirely to the chow diet. Presumably, differences in the mode of transmission of the effect across generations account for the differential phenotypes in F_2_ and F_3_ generation rats. It is notable that we did not find any body weight effect in the F_1_ generation, which is inconsistent with our previous studies showing increased body weight in F_1_-A1221 rats [12, 13]. We are unsure of the reason for this discrepancy, but small variances in environmental housing conditions, food lots, or other factors such as the microbiota that change the rats’ external and internal environment must be considered.

The finding of increased body weight is consistent with the “obesogen hypothesis,” which suggests that environmental factors including EDCs are contributing factors to the increasing incidence of obesity and metabolic syndrome [43–46]. Increased adipogenesis, disruption of insulin and glucose regulation [47–50], and specific alterations in lipid metabolism [51, 52], have also been reported in association with PCBs and other EDCs. Furthermore, a transgenerational phenotype was shown for another class of EDCs, tributyltin [16] and a mixture of EDCs in plastics [14].

In the current study, A1221 did not cause overt obesity, but resulted in a 5–10% increase in body weight. Notably, birth outcomes (numbers of pups, number of resorption sites on the uterus, and sex ratio) were unaffected in the PCB lineages. Therefore, it is unlikely that maternal-fetal interactions affecting energy balance related to intrauterine growth would underlie this body weight difference. Furthermore, litters were equalized to 3 males and 3 females at birth, so nursing demands on the dam were equivalent across the study [53]. Future work should confirm the A1221-induced, transgenerational phenotype on body weight, and secondly, investigate molecular (epigenetic) underpinnings of the adult phenotype and its maternal lineage-specific transmission. In this context, investigations into a potential role of sex-specific effects on primordial germ cell (PGC) de- and re-methylation dynamics are especially warranted, as female embryonic PGCs, at least in the mice, remain demethylated for longer (E16.5) than male embryonic PGCs (E13.5) [38]. Thus, while comparative DNA methylation dynamics have not been described for rat PGCs, the increases in body weight specific to offspring of the maternal lineage may be a consequence of the temporal overlap of A1221 exposure and the onset of DNA remethylation in female, but not male PGCs.

As a whole, the current model provides an environmentally realistic and timely opportunity to consider the integration of different hypotheses of the etiology of obesity and metabolic disease over generational timeframes, namely, the obesogen hypothesis [43–46], the DOHAD hypothesis [30–32], and the life-style hypothesis, the latter postulating diet and sedentary lifestyles as important factors of metabolic disease [54]. This is particularly relevant in order to delineate the interaction of ancestral burdens of previously unregulated contaminants, such as PCBs, and their interaction with additional environmental challenges to energy homeostasis. Although the body weight phenotype of the F_2_ and F_3_ generation was an unexpected finding of current work and the study was not designed to get at metabolic mechanisms, this is something we hope to pursue in the future.

Although there were small effects of A1221 and EB across generations on the timing of vaginal opening/preputial separation, and on anogenital index, the most interesting effects were on serum hormone concentrations in adulthood. In F_2_ females, serum progesterone concentrations were significantly lower in A1221 than control rats, regardless of lineage, while conversely, and specific to the F_3_-maternal lineage females, serum progesterone in A1221 rats were increased compared to DMSO and EB. For serum estradiol, F_3_ A1221 females had significantly higher concentrations compared to DMSO controls, irrespective of lineage. Together, these findings reveal that circulating sex steroids have unique alterations in females of the F_2_ and F_3_ generations descended from the A1221 lineage. Consistent with this present finding, a previous study from our lab [4] demonstrated significantly reduced circulating progesterone concentrations in F_2_ females compared to DMSO controls. It is also interesting that the hormonal phenotype did not emerge until the F_2_ (progesterone) or F_3_ (estradiol, progesterone) generations. Although further research is needed to provide a mechanistic understanding, we speculate that the exposure of the F_2_ generation as germ cells affected the programming of gonadal or neuroendocrine cells responsible for hormone synthesis and release, and that through germline transmission was manifested as an emergent phenotype in the F_3_ generation.

## Conclusions

As a whole, our study shows that ancestral exposure to A1221 caused sex- and lineage-specific inter- and transgenerational effects. It is not surprising that the sexes had a unique phenotype, as the mechanisms by which hormones affect brain and somatic development, the timing of germline development, and epigenetic mechanisms such as DNA methylation, histone modifications, and small non-coding RNAs, differ in timing between the sexes [20, 39, 40]. In a companion study, we are evaluating expression of suites of genes in the brains of these animals in order to evaluate neuroendocrine functional and underlying molecular changes that may contribute to the body weight and hormonal outcomes.

These results have important implications for humans. We are now in the 3rd generation since the Chemical Revolution of the mid-twentieth century, and even banned chemicals such as PCBs have a persistent imprint on the health of our descendants. There is no doubt that the same epigenetic mechanisms that are responsible for transgenerational effects in rodents are conserved in humans [55]. While best-studied for dietary factors (over- or under-nutrition), the heritability of the propensity to develop metabolic or endocrine disease across generations has been reported in humans, albeit mainly only up to the F_2_ generation [56, 57]. Moreover, the population of humans descending from women exposed in utero to the pharmaceutical estrogen, diethylstilbestrol, has been followed to the F_2_ generation, with increased reproductive abnormalities such as hypospadias in sons [58] and birth defects in both sexes, especially heart conditions [59] reported. Those populations are being followed, with results on the F_3_ generation forthcoming. Thus, ancestral exposure to environmental chemicals and pharmaceuticals has the potential to affect the propensity for health and disease for generations to come.

## Additional files

Additional file 1: **Figure S1.** Birth outcomes are shown for: (**A**) litter size, (**B**) resorption numbers, determined by counting implantation sites on the uterus post-mortem and comparing to the number of live births, and (**C**) sex ratio (male: female). Individual litter datapoints are shown for each bar. Data were analyzed by Kruskal-Wallis test. No significant differences were detected. (EPS 1304 kb)

Additional file 2: **Figure S2.** Estrous cyclicity was evaluated daily beginning on the day of vaginal opening through euthanasia in females. Shown are: (**A**) Number of estrous cycles, (**B**) average cycle length in days, and (**C**) percentage of elongated + irregular estrous cycles. Data were analyzed by Kruskal-Wallis test. No significant treatment effects were observed. (EPS 930 kb)

Additional file 3: **Figure S3.** General linear model analysis results of developmental, morphometric and endocrine endpoints analyzed in this study. (PDF 133 kb)

## Abbreviations

A1221: Aroclor 1221
AGD: Anogenital distance
AGI: Anogenital index
DMSO: Dimethylsulfoxide (vehicle control)
DOHAD: Developmental origins of health and disease
E: Embryonic day of age
EB: Estradiol benzoate
EDC: Endocrine-disrupting chemical
ER: Estrogen receptor
P: Postnatal day of age
PCB: Polychlorinated biphenyl
