# Supplementary material for: Transgenerational effects of polychlorinated biphenyls: 1. Development and physiology across 3 generations of rats
Source: Environ Health. 2018 Feb 20;17:18. doi: 10.1186/s12940-018-0362-5 (PMC5819226; doi:10.1186/s12940-018-0362-5)
Supplement: Supplementary file 3 — Figure S3. General linear model analysis results of developmental, morphometric and endocrine endpoints analyzed in this study. (PDF 133 kb) [file 12940_2018_362_MOESM3_ESM.pdf]

F1 BODYWEIGHT

Greenhouse-Geisser corrected			
Within subjects			
	**df**	**F**	**p value**
**time**	1.449	1265706	0.001
**time*litter**	1.449	0.819	0.586
**time*treatment**	2.899	1.982	0.114
**time*sex**	1.499	1226.553	0.001
**time*treatment*sex**	2.899	1.499	0.216
Between subjects			
	**df**	**F**	**p value**
**Litter**	1	2.935	0.088
**Treatment**	2	1.564	0.212
**Sex**	1	583.253	0.001
**Treatment*Sex**	2	1.693	0.187

F2 BODYWEIGHT

Greenhouse-Geisser corrected			
Within subjects			
	**df**	**F**	**p value**
**time**	1.415	19144.429	0.01
**time*litter**	1.415	2.388	0.11
**time*treatment**	2.83	7.765	0.001
**time*sex**	1.415	2418.686	0.001
**time*lineage**	1.415	8.913	0.001
**time*treatment*sex**	2.83	0.827	0.474
**time*treatment*lineage**	2.83	3.412	0.019
**time*sex*lineage**	1.415	3.112	0.063
**time*treatment*sex*lineage**	2.83	0.241	0.857
**Post-hoc Time*Treatment (Sidak)**	a	b	c
**P7**	DMSO,EB	DMSO,A1221	
**P14**	DMSO,EB	DMSO,A1221	
**P21**	DMSO,EB	DMSO,A1221	
**P28**	DMSO,EB	A1221	
**P35**	DMSO,EB	A1221	
**P42**	DMSO,EB	A1221	
**P49**	DMSO,EB	A1221	
**P56**	DMSO,EB	A1221	
**Post-hoc Time*Treatment*Lineage (Sidak)**	a	b	c
**P1 maternal**	DMSO,EB	A1221	
**P7 maternal**	DMSO,EB	DMSO, A1221	

**P21 maternal**	DMSO,EB	DMSO, A1221	
**P28 maternal**	DMSO,EB	A1221	
**P35 maternal**	DMSO,EB	A1221	
**P42 maternal**	DMSO,EB	A1221	
**P49 maternal**	DMSO,EB	A1221	
**P56 maternal**	DMSO,EB	A1221	
Between subjects			
	**df**	**F**	**p value**
**Litter**	1	7.325	0.007
**Treatment**	2	9.305	0.001
**Sex**	1	1198.714	0.001
**Lineage**	1	24.478	0.001
**Treatment*Sex**	2	1.029	0.358
**Treatment*Lineage**	2	3.65	0.027
**Sex*Lineage**	1	1.773	0.184
**Treatment*Sex*Lineage**	2	0.231	0.794
**Post-hoc Treatment (Sidak)**	a	b	c
**Treatment**	DMSO, EB	A1221	
**Post-hoc Treatment*lineage(Sidak)**	a	b	c
**Maternal**	DMSO,EB	A1221	

F3 BODYWEIGHT

**Greenhouse-Geisser corrected**			
Within subjects			
	**df**	**F**	**p value**
**time**	1.417	14589.9	0.001
**time*litter**	1.417	0.572	0.507
**time*treatment**	2.835	6.587	0.001
**time*sex**	1.417	1849.885	0.001
**time*lineage**	1.417	21.487	0.001
**time*treatment*sex**	2.835	1.432	0.234
**time*treatment*lineage**	2.835	3.487	0.019
**time*sex*lineage**	1.417	1.174	0.297
**time*treatment*sex*lineage**	2.835	1.06	0.857
**Post-hoc Time*Treatment (Sidak)**	a	b	c
**P35**	DMSO,EB	DMSO,A1221	
**P42**	DMSO,EB	DMSO,A1221	
**P49**	DMSO	EB	A1221
**P56**	DMSO	EB	A1221
**Post-hoc Time*Treatment*Lineage (Sidak)**	a	b	c
**P42 maternal**	DMSO,EB	A1221	

**P49 maternal**	DMSO,EB	A1221	
**P56 maternal**	DMSO,EB	A1221	
Between subjects			
	**df**	**F**	**p value**
**Litter**	1	0.116	0.088
**Treatment**	2	6.217	0.002
**Sex**	1	1059.127	0.001
**Lineage**	1	45.625	0.001
**Treatment*Sex**	2	1.287	0.277
**Treatment*Lineage**	2	2.199	0.112
**Sex*Lineage**	1	1.186	0.277
**Treatment*Sex*Lineage**	2	1.261	0.285
**Post-hoc Treatment (Sidak)**	a	b	c
Treatment	DMSO, A1221	EB	

F1 EYE OPENING

Between subjects			
	**df**	**H**	**p value**
**Litter**	1	1.409	0.237
**Treatment**	2	1.172	0.312
**Sex**	1	4.494	0.035

F2 EYE OPENING

Between subjects			
	**df**	**H**	**p value**
**Litter**	1	0.912	0.34
**Treatment**	2	6.89	0.001
**Sex**	1	3.921	0.048
**Lineage**	1	0.241	0.623
**Treatment (Dunn)**	a	b	c
**Eye opening**	DMSO,EB	DMSO,A1221	

F3 EYE OPENING

Between subjects			
	**df**	**H**	**p value**
**Litter**	1	2.035	0.155
**Treatment**	2	2.653	0.072
**Sex**	1	3.235	0.073
**Lineage**	1	0.173	0.677

F1 PUBERTY

Between subjects			
	**df**	**H**	**p value**
**Litter**	1	12.581	0.001
**Treatment**	2	0.784	0.458
**Sex**	1	659.479	0.001

F2 PUBERTY

Between subjects			
	**df**	**H**	**p value**
**Litter**	1	23.645	0.001
**Treatment**	2	5.435	0.005
**Sex**	1	1421.569	0.001
**Lineage**	1	20.435	0.001
**Treatment (Sidak)**	a	b	c
**Puberty**	DMSO,EB	DMSO, A1221	

F3 PUBERTY

Between subjects			
	**df**	**H**	**p value**
**Litter**	1	6.758	0.001
**Treatment**	2	4.427	0.013
**Sex**	1	1181.176	0.001
**Lineage**	1	3.161	0.076
**Treatment (Sidak)**	a	b	c
**Puberty**	DMSO,EB	DMSO,A1221	

F1 AGI

Greenhouse-Geisser corrected			
Within subjects			
	**df**	**F**	**p value**
**time**	1.449	366.327	0.001
**time*litter**	1.449	13.814	0.001
**time*treatment**	2.899	1.607	0.178
**time*sex**	1.499	68.138	0.001
**time*treatment*sex**	2.899	0.528	0.697
Between subjects			
	**df**	**F**	**p value**
**Litter**	1	2.895	0.001
**Treatment**	2	9.551	0.003
**Sex**	1	226.893	0.001
**Treatment*Sex**	2	0.011	0.685
**Post-hoc Time*Treatment (Sidak)**	a	b	c
**AGI**	DMSO,A1221	EB, A1221	

F2 AGI

Greenhouse-Geisser corrected			
Within subjects			
	**df**	**F**	**p value**
**time**	1.415	561.299	0.001
**time*litter**	1.415	9.07	0.001
**time*treatment**	2.83	2.286	0.067
**time*sex**	1.415	43.085	0.001
**time*lineage**	1.415	11.374	0.001
**time*treatment*sex**	2.83	1.859	0.125
**time*treatment*lineage**	2.83	1.903	0.114
**time*sex*lineage**	1.415	2.489	0.091
**time*treatment*sex*lineage**	2.83	0.241	0.857
Between subjects			
	**df**	**F**	**p value**
**Litter**	1	0.047	0.829
**Treatment**	2	2.12	0.145
**Sex**	1	1052.453	0.001
**Lineage**	1	22.871	0.001
**Treatment*Sex**	2	1.291	0.276
**Treatment*Lineage**	2	2.161	0.117
**Sex*Lineage**	1	1.206	0.273
**Treatment*Sex*Lineage**	2	1.235	0.292

F3 AGI

Greenhouse-Geisser corrected			
Within subjects			
	df	F	p value
**time**	1.649	295.596	0.001
**time*litter**	1.649	46.373	0.001
**time*treatment**	0.046	0.635	0.607
**time*sex**	1.64	163.981	0.001
**time*lineage**	1.649	23.773	0.001
**time*treatment*sex**	0.083	1.148	0.331
**time*treatment*lineage**	0.257	3.571	0.011
**time*sex*lineage**	0.002	0.048	0.927
**time*treatment*sex*lineage**	0.083	1.16	0.326
**Post-hoc Time*Treatment*Lineage (Sidak)**	a	b	c
P7 maternal	DMSO	EB	A1221
P14 maternal	DMSO	EB	A1221
Between subjects			
	df	F	p value
**Litter**	1	22.976	0.001
**Treatment**	2	0.858	0.425
**Sex**	1	11713.594	0.001
**Lineage**	1	7.263	0.007
**Treatment*Sex**	2	0.906	0.405
**Treatment*Lineage**	2	3.368	0.035
**Sex*Lineage**	1	0.529	0.468
**Treatment*Sex*Lineage**	2	0.515	0.598
**Post-hoc Treatment*Lineage (Sidak)**	a	b	c
Maternal	DMSO,EB	EB,A1221	

F1 GONADOSOMATIC INDEX

Between subjects			
	**df**	**F**	**p value**
**Litter**	1	0.516	0.474
**Treatment**	2	0.021	0.979
**Sex**	1	13557.736	0.001
**Treatment*Sex**	2	0.085	0.919

F1 UTERINE INDEX

Between subjects			
	**df**	**F**	**p value**
**Litter**	1	2.189	0.144
**Treatment**	2	0.936	0.395

F1 ADRENAL INDEX

Between subjects			
	**df**	**F**	**p value**
**Litter**	1	6.666	0.011
**Treatment**	2	0.936	0.395
**Sex**	1	788.255	0.001
**Treatment*Sex**	2	0.559	0.573

F2 GONADOSOMATIC INDEX

Between subjects			
	**df**	**F**	**p value**
**Litter**	1	0.855	0.356
**Treatment**	2	0.052	0.949
**Sex**	1	32341.379	0.001
**Lineage**	1	1.556	0.214
**Treatment * Sex**	2	0.012	0.988
**Treatment * Lineage**	2	0.506	0.603
**Sex * Lineage**	1	2.668	0.104
**Treatment * Sex * Lineage**	2	0.775	0.462

F2 UTERINE INDEX

Between subjects			
	**df**	**F**	**p value**
**Litter**	1	0.434	0.511
**Treatment**	2	0.395	0.675
**Lineage**	1	0.045	0.832
**Treatment * Lineage**	2	0.56	0.573

F2 Adrenal index

Between subjects			
	**df**	**F**	**p value**
**Litter**	1	0.189	0.664
**Treatment**	2	0.279	0.757
**Sex**	1	231.215	0.001
**Lineage**	1	1.475	0.226
**Treatment * Sex**	2	0.549	0.578
**Treatment * Lineage**	2	0.016	0.984
**Sex * Lineage**	1	0.245	0.621
**Treatment * Sex * Lineage**	2	1.095	0.336

F3 GONADOSOMATIC INDEX

Between subjects			
	**df**	**F**	**p value**
**Litter**	1	0	0.997
**Treatment**	2	0.068	0.934
**Sex**	1	31329.201	0.001
**Lineage**	1	2.297	0.131
**Treatment * Sex**	2	0.114	0.892
**Treatment * Lineage**	2	2.786	0.064
**Sex * Lineage**	1	2.083	0.15
**Treatment * Sex * Lineage**	2	2.63	0.074

F3 UTERINE INDEX

Between subjects			
	**df**	**F**	**p value**
**Litter**	1	0.197	0.658
**Treatment**	2	0.207	0.813
**Lineage**	1	0.256	0.614
**Treatment * Lineage**	2	1.475	0.232

F3 Adrenal index

Between subjects			
	**df**	**F**	**p value**
**Litter**	1	5.379	0.021
**Treatment**	2	0.426	0.654
**Sex**	1	1602.447	0.001
**Lineage**	1	13.048	0.001
**Treatment * Sex**	2	0.781	0.459

**Treatment * Lineage**	2	2.024	0.134
**Sex * Lineage**	1	7.734	0.006
**Treatment * Sex * Lineage**	2	2.21	0.112

F1 ESTRADIOL

Between subjects			
	**df**	**F**	**p value**
**Litter**	1	11.333	0.001
**Treatment**	2	1.049	0.354
**Sex**	1	54.76	0.001
**Treatment*Sex**	2	1.429	0.244

F1 PROGESTERONE

Between subjects			
	**df**	**F**	**p value**
**Litter**	1	0.293	0.591
**Treatment**	2	1.999	0.147
**Sex**	1	867.459	0.001
**Treatment*Sex**	2	0.108	0.898

F1 TESTOSTERONE (MEASURED IN MALES ONLY)

Between subjects			
	**df**	**F**	**p value**
**Litter**	1	0.005	0.946
**Treatment**	2	0.061	0.941

F1 CORTISONE

Between subjects			
	**df**	**F**	**p value**
**Litter**	1	0.422	0.519
**Treatment**	2	0.011	0.989
**Sex**	1	36.964	0.001
**Treatment*Sex**	2	0.414	0.663

F2 ESTRADIOL

Between subjects			
**Litter**	1	6.61	0.011
**Treatment**	2	0.406	0.667
**Sex**	1	6.932	0.009
**Lineage**	1	2.54	0.112
**Treatment * Sex**	2	0.721	0.487
**Treatment * Lineage**	2	0.996	0.371
**Sex * Lineage**	1	0.089	0.765
**Treatment * Sex * Lineage**	2	0.854	0.427

F2 Progesterone

Between subjects			
	**df**	**F**	**p value**
**Litter**	1	2.688	0.104
**Treatment**	2	3.32	0.04
**Sex**	1	173.632	0.001
**Lineage**	1	0.906	0.344
**Treatment * Sex**	2	3.517	0.033
**Treatment * Lineage**	2	1.078	0.344
**Sex * Lineage**	1	0.74	0.392
**Treatment * Sex * Lineage**	2	0.958	0.387
**Posthoc Treatment*Sex (Sidak)**	a	b	c
Female	DMSO,EB	EB,A1221	

F2 TESTOSTERONE (MEASURED IN MALES ONLY)

Between subjects			
	**df**	**F**	**p value**
**Litter**	1	0.221	0.64
**Treatment**	2	1.195	0.311
**Lineage**	1	0.299	0.587
**Treatment * Lineage**	2	0.46	0.634

F2 CORTISONE

Between subjects			
**Litter**	1	5.103	0.026
**Treatment**	2	0.531	0.59
**Sex**	1	81.287	0.001
**Lineage**	1	0.463	0.497
**Treatment * Sex**	2	1.105	0.335
**Treatment * Lineage**	2	0.111	0.895
**Sex * Lineage**	1	2.377	0.126
**Treatment * Sex * Lineage**	2	2.692	0.072

F3 ESTRADIOL

Between subjects			
**Litter**	1	0.022	0.882
**Treatment**	2	3.329	0.037
**Sex**	1	65.861	0.001
**Lineage**	1	1.413	0.236
**Treatment * Sex**	2	5.142	0.007
**Treatment * Lineage**	2	0.084	0.919

**Sex * Lineage**	1	1.293	0.257
**Treatment * Sex * Lineage**	2	0.782	0.459
**Posthoc Treatment*Sex (Sidak)**	a	b	c
**Female**	DMSO,EB	A1221	

F3 Progesterone

Between subjects			
	**df**	**F**	**p value**
**Litter**	1	0.088	0.767
**Treatment**	2	2.245	0.111
**Sex**	1	265.839	0.001
**Lineage**	1	6.686	0.011
**Treatment * Sex**	2	2.272	0.108
**Treatment * Lineage**	2	3.27	0.042
**Sex * Lineage**	1	6.673	0.011
**Treatment * Sex * Lineage**	2	3.229	0.044
**Posthoc Treatment*Lineage (Sida**	a	b	c
**Maternal**	DMSO,EB	EB,A1221	
**Posthoc Treatment*Lineage*Sex (**	a	b	c
**Maternal Female**	DMSO,EB	A1221	

F3 TESTOSTERONE (MEASURED IN MALES ONLY)

Between subjects			
	**df**	**F**	**p value**
**Litter**	1	2.453	0.123
**Treatment**	2	1.995	0.146
**Lineage**	1	2.741	0.104
**Treatment * Lineage**	2	1.18	0.315

F3 CORTISONE

Between subjects			
**Litter**	1	0.891	0.347
**Treatment**	2	1.968	0.145
**Sex**	1	72.316	0.001
**Lineage**	1	0.215	0.644
**Treatment * Sex**	2	0.076	0.927
**Treatment * Lineage**	2	0.558	0.574
**Sex * Lineage**	1	0.134	0.715
**Treatment * Sex * Lineage**	2	0.524	0.594

F1 NUMBER OF ESTROUS CYCLES

Between subjects			
	**df**	**H**	**p value**
**Litter**	36	38.27	0.368
**Treatment**	2	4.49	0.11

F1 AVERAGE CYCLE LENGTH

Between subjects			
	**df**	**H**	**p value**
**Litter**	36	52.91	0.034
**Treatment**	2	0.671	0.715

F1 NUMBER OF ELONGATED AND IRREGULAR CYCLES

Between subjects			
	**df**	**H**	**p value**
**Litter**	36	44.81	0.149
**Treatment**	2	0.369	0.789

F2 NUMBER OF ESTROUS CYCLES

Between subjects			
	**df**	**H**	**p value**
**Litter**	78	91.869	0.041
**Treatment**	2	4.505	0.18
**Lineage**	1	0.324	0.57

F2 AVERAGE CYCLE LENGTH

Between subjects			
	**df**	**H**	**p value**
**Litter**	78	117.791	0.001
**Treatment**	2	5.073	0.312
**Lineage**	1	0.24	0.217

F2 NUMBER OF ELONGATED AND IRREGULAR CYCLES

Between subjects			
	**df**	**H**	**p value**
**Litter**	78	90.61	0.049
**Treatment**	2	4.693	0.151
**Lineage**	1	1.825	0.178

F3 NUMBER OF ESTROUS CYCLES

Between subjects

	df	H	p value
**Litter**	70	95.515	0.016
**Treatment**	2	0.518	0.772
**Lineage**	1	0.16	0.689

F3 AVERAGE CYCLE LENGTH

Between subjects			
	**df**	**H**	**p value**
**Litter**	70	92.858	0.024
**Treatment**	2	0.356	0.837
**Lineage**	1	2.366	0.124

F3 NUMBER OF ELONGATED AND IRREGULAR CYCLES

Between subjects			
	**df**	**H**	**p value**
**Litter**	70	71.713	0.356
**Treatment**	2	0.637	0.727
**Lineage**	1	0.004	0.947
